# Etanercept-Induced Myelopathy in a Pediatric Case of Blau Syndrome

**DOI:** 10.1155/2011/134106

**Published:** 2012-01-15

**Authors:** Fabiola Caracseghi, Jaume Izquierdo-Blasco, Angel Sanchez-Montanez, Susana Melendo-Perez, Manuel Roig-Quilis, Consuelo Modesto

**Affiliations:** ^1^Pediatric Rheumatology Department, Vall d'Hebron University Hospital, P. Vall d'Hebron 119-129, 08035 Barcelona, Spain; ^2^Pediatric Neurology Department, Vall d'Hebron University Hospital, P. Vall d'Hebron 119-129, 08035 Barcelona, Spain; ^3^Pediatric Radiology Department, Vall d'Hebron University Hospital, P. Vall d'Hebron 119-129, 08035 Barcelona, Spain; ^4^Pediatric Emergency Department, Vall d'Hebron University Hospital, P. Vall d'Hebron 119-129, 08035 Barcelona, Spain

## Abstract

Blau syndrome is a rare autoinflammatory disorder within the group of pediatric granulomatous diseases. Mutations in nucleotide-binding oligomerization domain 2 (NOD2/CARD15) are responsible for this condition, which has an autosomal dominant pattern of inheritance and variable expressivity. The clinical picture includes arthritis, uveitis, skin rash, and granulomatous inflammation. Central nervous system involvement is seldom reported, although some isolated cases of seizures, neurosensorial hearing loss, and transient cranial nerve palsy have been described. Treatment consists of nonsteroidal anti-inflammatory drugs, corticosteroids, and immunosuppressive agents, among which anti-tumor-necrosis-factor-alpha (TNF-**α**) biologic agents, such as etanercept, play an important role. Among the major adverse effects of TNF-**α** inhibitors, demyelinating disease, multiple sclerosis, and acute transverse myelitis have been reported in adults. We describe a case of pediatric Blau syndrome affected by etanercept-induced myelopathy, manifesting as a clinical syndrome of transverse myelitis. The patient experienced rapid recovery after etanercept was discontinued. To our knowledge, this is the first such case reported in the literature and, possibly, the one with the latest onset, following 8 years of treatment. We discuss the etiopathogenic mechanisms of this reaction and possible explanations for the imaging findings.

## 1. Introduction

Blau syndrome is a rare autoinflammatory disorder within the group of pediatric granulomatous diseases, together with early-onset sarcoidosis [1, 2]. Mutations in nucleotide-binding oligomerization domain 2 (NOD2/CARD15), a member of the NOD-like receptor family of intracellular proteins, are responsible for the disease, which has an autosomal dominant pattern of inheritance and variable expressivity.

The clinical picture includes arthritis, uveitis, skin rash, and granulomatous inflammation [1, 3]. Central nervous system (CNS) involvement is seldom reported, although isolated cases of seizures, neurosensorial hearing loss and transient cranial nerve palsy have been described [4]. Fever and acute-phase reaction are not always present [2, 3].

Treatment consists of nonsteroidal anti-inflammatory drugs, corticosteroids, and, in refractory cases, immunosuppressive agents, such as methotrexate, azathioprine, mycophenolate mofetil and, recently, interleukin-1 blockers (anakinra), and anti-tumor-necrosis-factor-alpha (TNF-*α*) biologic agents. Anti-TNF-*α* drugs, such as etanercept, infliximab, and adalimumab have been on the market since 1998. Etanercept, a soluble recombinant dimer of human TNF receptor proteins fused and bound to human IgG_1_, acts competitively to inhibit TNF binding to its cell surface receptor. Infliximab and adalimumab are monoclonal anti-TNF-*α* antibodies, the first a murine chimeric and the latter a humanized antibody [3].

Anti-TNF-*α* treatment has been successfully used for several autoimmune and autoinflammatory conditions, such as rheumatoid arthritis, psoriasis with or without arthritis, ankylosing spondylitis, juvenile idiopathic arthritis, and Crohn's disease. Because of the low prevalence of Blau syndrome, there is little information on anti-TNF-*α* use in pediatric patients with this disease.

The major adverse effects of TNF-*α* inhibitors include local injection site and systemic reactions after intravenous infusion, infections (particularly opportunistic, due to fungi and mycobacteria), lymphoproliferative diseases, and systemic lupus erythematosus-like syndromes. Demyelinating diseases, multiple sclerosis, and acute transverse myelitis have also been reported in adults [5].

We describe the case of a pediatric patient with Blau syndrome affected by etanercept-induced myelopathy, manifesting as a clinical syndrome of transverse myelitis. To our knowledge, this is the first such case reported in the literature. A distinctive feature was its late onset, 8 years after the start of treatment.

## 2. Case Presentation

A 13-year-old boy presented to the emergency unit with inability to stand or walk. Eight days previously, he had experienced a mild coccygeal trauma while playing soccer. Seven days later he presented paresthesia of the lower limbs and, less than 24 hours later, bilateral hypoesthesia and paraparesis. He was unable to initiate urination or defecation, but was not incontinent. He denied fever and any infectious episodes over the previous weeks.

The patient had been diagnosed of Blau syndrome at the age of 5. The condition manifested as a generalized papulous rash, recurrent arthritis, and tenosynovitis, which started when he was 2 years old. His mother had been misdiagnosed as having rheumatoid arthritis as a child, after presenting similar symptoms. Genetic study confirmed an autosomal dominant mutation in the NOD2/CARD15 gene. The patient had been treated earlier with corticosteroids and methotrexate and, over the previous 8 years, since the diagnosis, had also received etanercept, with good disease control. He had never presented ocular manifestations.

Physical examination revealed a normal mental status, with no cranial nerve involvement. Funduscopic examination was normal. Muscle tone strength and deep tendon reflexes of the upper limbs were normal. He had hyperreflexia in both lower limbs, an extensor plantar reflex and bilateral exhaustible clonus. Muscle strength in the lower limbs was decreased, graded 2 to 4 out of a maximum of 5 in the different muscle groups, the most highly affected being the psoas and quadriceps. He had tactile and pain hypoesthesia with a sensitive level at T12 and normal thermal and vibratory sensation. Painless camptodactyly and flexion contractures of the proximal interphalangeal joints of the fourth and fifth fingers had already been documented, and there were no inflamed joints. He had no spleen or liver enlargement and no acute skin lesions. The remainder of the examination was normal.

Blood analyses were unremarkable, except for a high erythrocyte sedimentation rate (85 mm/h, normal value <10 mm/h). Cerebrospinal fluid glucose level was normal, protein was slightly elevated (78 mg/dL, normal value 15–45 mg/dL), and IgG level was high (7.4 mg/dL, normal value <3.4 mg/dL), without pleocytosis or oligoclonal bands. Bacterial, viral, and fungal microbiological tests were negative, including mycobacteria.

**Figure 1:**
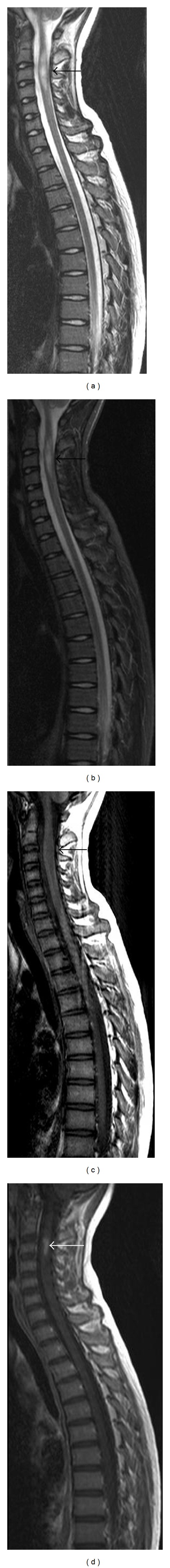
MR images of the cervicodorsal spinal cord. Sagittal T2WI (a) and STIR-T2WI (b) show a well-defined, ovoid-shaped, high-intensity lesion (black arrow) within the cervical cord at C_2_–C_4_, with mild fusiform expansion of the cord. On sagittal T1WI (c), the lesion is slightly hypointense (black arrow). After gadolinium administration (d), the lesion enhances intensely and homogeneously (white arrow).

**Figure 2:**
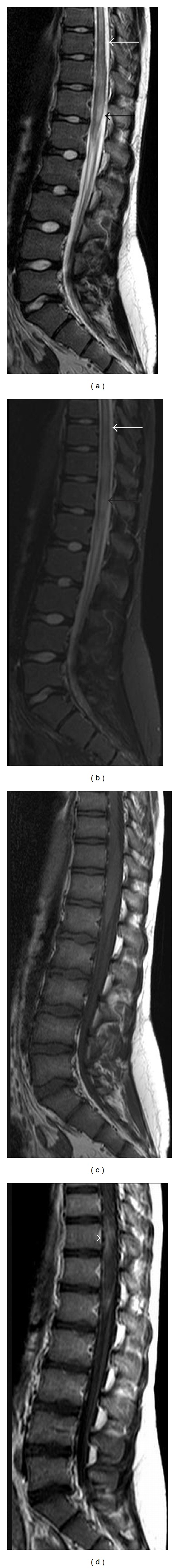
MR images of the dorsolumbar spinal cord. Sagittal T2WI (a) and STIR-T2WI (b) show patchy hyperintense lesions involving the distal lumbar spinal cord and the conus medullaris (black arrows). Mild spinal cord edema is also present (white arrows). Incidentally, posterior disc bulging is visualized at the level of L_5_-S_1_ (black arrowhead). The lesions are isointense on T1WI (c). T1WI after intravenous gadolinium injection (d) demonstrates heterogeneous enhancement (white arrowhead).

Cranial magnetic resonance imaging (MRI) study was normal. MRI of the spinal cord revealed bifocal high-intensity white matter lesions on T2-weighted images (T2WI), one extending from the second to the fourth cervical (C_2_–C_4_) level and another at the conus medullaris (T_12_-L_1_), which enhanced after contrast administration (Figures 1 and 2). Diffusion-weighted imaging (DWI) and apparent diffusion coefficient maps failed to show water movement restriction. 

 Considering the previous reports of similar adverse reactions with anti-TNF-*α* [5–12], etanercept was discontinued. The patient received intravenous methylprednisolone pulse therapy (1 g/day) during 5 days, followed by oral prednisone starting at 1 mg/kg, with slow dose tapering for 6 weeks. Clinical improvement was noted 36 hours after the first infusion, and the patient was able to walk without help and regain sphincter control 5 days later. Mild proximal muscle weakness, hyperreflexia, and paresthesia persisted during the next 2 weeks, with progressive amelioration. Four weeks later, his recovery was nearly complete, with slight hyperreflexia in the lower limbs.

**Figure 3:**
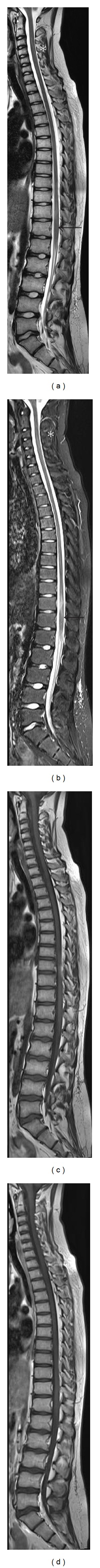
Whole cord MR images 6 weeks after the first MRI. Sagittal T2WI (a) and STIR-T2WI (b) show resolution of the cervical lesion (white asterisks), whereas a slight hyperintensity is seen in the lumbar cord (black arrows). There are no abnormalities on sagittal T1WI without (c) and with intravenous gadolinium (d).

Follow-up MRI performed 6 weeks after the first study revealed complete resolution of the cervical cord lesion and an improvement with no enhancement of the conus medullaris lesion (Figure 3). 

 At his last clinical follow-up visit, 3 months after onset, the patient was completely symptom-free, and the neurological examination was normal. His baseline treatment was switched to corticosteroids and methotrexate. New biological drugs will be reconsidered depending on his disease control, but anti-TNF-*α* agents are excluded.

## 3. Discussion

The patient exhibited a clinical syndrome of acute transverse myelitis. The mild coccygeal trauma he had sustained previously may have acted as a triggering event, as has been previously reported [6]. Although etanercept undoubtedly played a role in this patient's myelopathy, the underlying mechanisms are difficult to establish, especially in the absence of pathological studies. It is noteworthy that imaging study revealed 2 lesions at different levels, but the cervical lesion showed no clinical expression on detailed neurological examination and the clinical and MRI manifestations progressively disappeared over a 4- to 6-week period.

The shape of the lesions, the lateral position within the spinal cord, and the enhancement suggest demyelination as the most plausible origin of the patient's MRI findings. MRI studies in cases of anti-TNF-*α*-associated complications reported to date usually show single or multiple lesions on T2WI, attributed to demyelination [5, 7]. As the cervical lesion completely disappeared 6 weeks after drug withdrawal and there are no previous reports of histological confirmation, other potential explanations should be considered.

Osmotic demyelination, defined as acute demyelination without inflammation, attributed to osmotic stress or rapid shifts in serum osmolality (classically due to rapid correction of hyponatremia), can produce a similar MRI pattern. The imaging findings may be due to a focal disruption of the brain blood barrier, leading to extravasation and transudation of fluid and macromolecules through arteriolar walls, similarly to what occurs in posterior reversible encephalopathy syndrome. Nonetheless, enhancement following gadolinium administration is not a common feature in these conditions. The imaging characteristics are also consistent with small-vessel lesion due to vasculitis or ischemia. However, although an ischemic etiology cannot be ruled out, the absence of ventral cord predilection and the lack of restriction on DWI make this possibility less likely. Lastly, infectious diseases of the spine can have a similar MRI appearance, but the clinical scenario rules out this possibility.

TNF-expressing macrophages are the effector cells responsible for active demyelination and axonal injury [5]. TNF-*α* is an important proinflammatory cytokine that is involved in the pathogenesis of inflammatory demyelination in many conditions [5]. It has heterogenic effects in the inflammatory cascade and immune regulation, some of which are not well understood and even antagonistic, depending on the type of receptors involved. For example, although it is an important cytokine implicated in neuronal inflammation, particularly when its type 1 receptor (TNFR1) is involved, TNF-*α* can also have a protective effect on the CNS and is a key element in remyelination through activation of its type 2 receptor (TNFR2) [5]. The effects of this cytokine on the CNS also depend on the underlying disease. A good example is multiple sclerosis, in which anti-TNF-*α* treatment worsens the disease, whereas in other conditions, such as chronic dysimmune neuropathies, it is successfully used to treat autoimmune demyelination.

The reported neurological complications related to anti-TNF-*α* drugs include visual disturbances, confusion, paresthesia, gait disturbances, ataxia, apraxia, dysarthria and even complete Miller-Fisher or Guillain-Barré syndromes, optic neuritis (unilateral or bilateral), pure sensory neuropathy, spasticity, neurogenic bladder, transverse myelitis, and seizures [5, 7, 8]. CNS demyelinating disease and peripheral neuropathy syndromes seem to be the most common [5].

Cerebrospinal fluid can be normal, but low glucose, high protein levels, pleocytosis (mononuclear or polynuclear), increased IgG, and the presence of oligoclonal bands have been reported [5–9].

Symptoms due to etanercept-related demyelination usually develop after a mean of 5 months of therapy [5], with a broad range, from 1 week to several years [5–10]. Our case is among those with the latest reported onset, occurring after 8 years of treatment.

It is difficult to establish an association between anti-TNF therapy and the development of demyelinating disease and peripheral neuropathy. There is, however, a temporal association between the use of these drugs and the development of symptoms, which improve after interrupting treatment and/or reappear after reexposure in most reported cases, as in ours. There are some factors against this association, such as the existence of an underlying condition, which could predispose to myelination defects (this is the case of multiple sclerosis, in which patients are at high risk of demyelination regardless of anti-TNF-*α* treatment) [5, 7]. Demyelinating lesions are not characteristic of Blau syndrome. Several theories have been put forth to explain this relation [5, 9], such as local immune dysregulation by TNF-*α* blockage leading to prolonged survival of myelin-specific activated T cells and increased cytokine production, vasculitis-induced ischemia and altered repairing of axonal injuries, and myelin damage by TNRF2 inhibition. But, up to now, long-term clinical studies assessing the safety of anti-TNF-*α* drugs have failed to show a higher rate of neurological disorders in treated patients than in the general population [5, 9, 11].

There is no specific treatment for this complication, but the immediate withdrawal of the causative drug seems reasonable, together with high-dose courses of corticosteroids, intravenous immunoglobulins (IVIG), or plasmapheresis [5–9]. The use of cyclophosphamide, interferon *β*-1a, azathioprine, or gabapentin, usually in association with steroids or IVIG, has also been reported, with variable results [5, 9]. In most cases, the prognosis is favorable, with partial or complete resolution of the symptoms (occurring spontaneously in some cases) and, sometimes, with persistent imaging abnormalities [5, 6]. Long-term followup has shown a variable course, suggesting that treatment withdrawal may not be necessary for disease control in individual patients who do not have disabling involvement and for whom there are no other available treatment options [10].

Cerebral demyelination possibly associated with etanercept treatment in children has been reported in the single case of a 5-year-old girl with systemic-onset juvenile idiopathic arthritis, but it was difficult to establish whether it was drug-related [12].

## 4. Conclusions

Anti-TNF therapy should be avoided in patients with a history of predisposing conditions, such as multiple sclerosis or peripheral neuropathy. Clinicians should carefully monitor their patients for new or unusual neurological symptoms appearing during treatment and discontinue therapy if there is a suspected drug-related complication. Based on the current experience, in individual cases, in which other treatment options are limited, anti-TNF drugs might be continued at a lower dose, under strict supervision.
